# Spatial transcriptomics reveals club cell features and strong CLDN18 expression along the mucinous pathway to invasive mucinous adenocarcinoma

**DOI:** 10.1002/path.70120

**Published:** 2026-09-08

**Authors:** Joon Young Park, Do Yeon Kim, Hyun Jung Lee, Jung Hee Lee, Hae Ryoun Park, Dong Hoon Shin

**Affiliations:** ^1^ Department of Pathology, School of Medicine Pusan National University Yangsan Republic of Korea; ^2^ Department of Oral Pathology Dental and Life Science Institute, School of Dentistry, Pusan National University Yangsan Republic of Korea

**Keywords:** invasive mucinous adenocarcinoma, lung adenocarcinoma, club cell, mucinous metaplasia, mucinous dysplasia

## Abstract

Invasive mucinous adenocarcinoma (IMA) of the lung is a distinct subtype of lung adenocarcinoma with unique clinicopathologic features; however, its histogenesis remains incompletely understood. Although mucinous metaplasia has been suspected as a precursor lesion, direct molecular evidence supporting this progression is limited. In this study, we applied morphology‐guided spatial transcriptomics to investigate epithelial lineage relationships across the mucinous spectrum of lung lesions. Formalin‐fixed, paraffin‐embedded lung tissues from 26 patients were analyzed using the GeoMx digital spatial profiling platform. A total of 118 cytokeratin‐positive regions of interest representing normal bronchioles, bronchiolar metaplasia, mucinous metaplasia, mucinous dysplasia, IMA, nonmucinous terminal respiratory unit–type adenocarcinoma, squamous metaplasia/dysplasia, and squamous cell carcinoma were profiled. Spatial transcriptomic data were integrated with two independent public cohorts and mapped to the Human Lung Cell Atlas for epithelial cell‐type assignment. Unsupervised clustering identified a distinct mucinous cluster comprising IMA and most mucinous dysplasia regions, characterized by enrichment of gastric‐type differentiation markers, including *MUC5AC*, *MUC6*, *TFF1*, *TFF2*, and *CLDN18*. Label‐transfer analysis demonstrated that mucinous metaplasia and dysplasia were predominantly associated with club and goblet cell identities, whereas IMAs showed enrichment of club and goblet signatures. In contrast, nonmucinous adenocarcinomas consistently aligned with alveolar type 2 cell identity across all datasets. Immunohistochemistry confirmed uniform CLDN18 expression in IMAs and its absence in nonmucinous adenocarcinomas. These findings provide spatial transcriptomic evidence supporting a club cell–associated mucinous pathway in lung tumorigenesis and identify CLDN18 as a defining molecular feature of IMA. © 2026 The Author(s). *The Journal of Pathology* published by John Wiley & Sons Ltd on behalf of The Pathological Society of Great Britain and Ireland.

## Introduction

A deeper understanding of premalignant biology and the putative cells of origin is essential for effective lung cancer interception. Defining the earliest cellular and molecular events enables the identification of high‐risk lesions and supports the development of biomarkers and therapeutic targets for early intervention [1]. Traditionally, nonmucinous adenocarcinomas have been considered to originate from club cells or type II pneumocytes [alveolar type 2 (AT2)] and designated as terminal respiratory unit (TRU)‐type adenocarcinomas [1, 2, 3]. In contrast, invasive mucinous adenocarcinoma (IMA) is recognized as a pathologically and clinically distinct subtype; however, its histogenesis remains controversial [4, 5]. Several groups, including ours, have proposed that IMA develops through a stepwise process involving mucinous metaplasia [6, 7, 8, 9]; however, the precise biological mechanisms driving this progression remain undefined. Morphologically, the mucous cells within the bronchiolar mucosa and honeycomb‐like cysts seen in interstitial lung disease closely mimic the features of IMA [7]. Moreover, adenocarcinomas arising from advanced lung fibrosis are often mucinous. Additionally, IMA is associated with bronchopulmonary dysplasia, a condition predominantly characterized by mucous cell proliferation [10]. Collectively, these observations support the hypothesis that mucous‐cell‐driven alterations play a central role in IMA histogenesis.

Recent advances in single‐cell research have enabled precise transcriptomic identification and classification of normal lung epithelial cell populations, including AT2, club, goblet, multi‐ciliated, and basal cells [11, 12]. Club cells are recognized as progenitors that produce terminally differentiated goblets and ciliated cells [11, 12]. The integration of computational frameworks with single‐cell technologies has allowed the systematic mapping of query cells onto well‐annotated reference datasets to infer cellular identity and origin [13, 14]. This approach also facilitates the comparison of tumor cells with their normal counterparts, thereby elucidating lineage relationships [15, 16]. Although single‐cell RNA sequencing (scRNA‐seq) is not easily applicable to formalin‐fixed, paraffin‐embedded tissues, emerging spatial transcriptomic platforms enable gene expression profiling of specific microscopic regions directly within tissue sections, allowing direct comparison with reference cell types [17]. In this study, we used the GeoMx spatial transcriptomics platform to evaluate gene expression patterns across a spectrum of lung lesions, including normal bronchioles (NB), bronchiolar metaplasia (BM), mucinous metaplasia (MM), a putative (hypothetical) mucinous dysplasia (MD), IMA, and nonmucinous TRU‐type adenocarcinoma. Squamous metaplasia frequently accompanies bronchiolization, and squamous dysplasia or squamous cell carcinoma may emerge from these lesions, so we also incorporated squamous metaplasia and dysplasia (SqM/D) and squamous cell carcinoma (SCC) into our analysis. Our objective was to clarify the relationships between these histological stages and compare their gene expression profiles with those of normal lung epithelial populations to better understand the histogenesis of IMA.

## Materials and methods

### Ethics approval and sample selection

The study was conducted according to the guidelines of the Declaration of Helsinki (https://www.wma.net/policies-post/wma-declaration-of-helsinki/) and approved by the Institutional Review Board of Pusan National University Yangsan Hospital (study no.: 55‐2025‐038). Twenty‐six formalin‐fixed, paraffin‐embedded tissue blocks were obtained from 26 patients who underwent surgical resection for lung cancer at Pusan National University Yangsan Hospital (Yangsan, Republic of Korea) between 2022 and 2025. From these, 119 regions of interest (ROIs) were selected for spatial transcriptomic profiling. The distribution of ROIs was balanced according to the study design and included 12 nonmucinous TRU‐type adenocarcinomas, 17 IMAs, 10 BMs, 16 MMs, 18 MDs, 15 SqM/Ds, 16 parenchymal SCCs (pSCCs), 12 central SCCs (cSCCs), and three NBs. Within each specimen, ROIs were defined based on morphologically representative regions, and multiple areas were selected within the same histological subtype to account for intratumoral heterogeneity.

### Spatial transcriptomics

Digital spatial profiling (DSP) was performed using the Whole Transcriptome Atlas (WTA) panel (NanoString, Seattle, WA, USA). Epithelial regions were identified using a pan‐cytokeratin antibody (PanCK, clone AE1/AE3; NanoString). Slides were scanned at ×20 magnification on the GeoMx DSP platform, and ROIs were manually selected based on histologically uniform areas (supplementary material, Figure S1). Libraries were prepared according to the manufacturer's protocol, pooled at equimolar concentrations prior to sequencing to normalize read depth across ROIs, and sequenced on an Illumina NovaSeq system. Raw FASTQ files were processed using the GeoMx NGS Pipeline to generate digital count (DCC) files. Probe‐level quality control (QC) excluded probes with a low probe ratio [(geomean across segments)/(geomean within target) ≤ 0.1] or those failing Grubbs’ outlier test in ≥ 20% of segments. At the segment level, the following QC thresholds were applied: minimum raw reads ≥ 1,000; percentage trimmed ≥ 80%; percentage stitched ≥ 80%; percentage aligned ≥ 80%; percentage saturation ≥ 50%; negative control counts ≥ 4; no‐template control counts ≤ 1,000; minimum nuclei ≥ 50; and minimum segment area ≥ 1,000 μm^2^. Probe counts were aggregated to the target level using the geometric mean and normalized by quantile normalization. The limit of detection (LOD) was defined as the negative control geometric mean plus two standard deviations, and genes detected above this threshold in at least 10% of ROIs were retained. Flagged ROIs were further evaluated by comparing the 75th percentile expression (Q3) to background signal [18], and one ROI from pSCC was excluded due to insufficient signal. Overall, 18,673 of 18,815 probes passed QC.

### Bioinformatic analyses

Before the analysis, two publicly available datasets, GSE200563 [19] and GSE265899 [20], were integrated using Seurat's FindIntegrationAnchors function. Spatial transcriptomic gene expression count matrices were analyzed using the Seurat R package. Principal component analysis was performed, and the top 20 principal components were used to construct a shared nearest neighbor graph and perform clustering at a resolution of 1.0. Cluster identification was performed using the FindMarkers function. The integrated dataset was visualized in a low‐dimensional space using Uniform Manifold Approximation and Projection (UMAP; RunUMAP function, default parameters) and subjected to copy number variation (CNV) analysis using the inferCNV R package. Finally, the integrated data were mapped to the Human Lung Cell Atlas (HLCA) as a reference for label transfer [11].

### Immunohistochemistry and *in situ* hybridization

Immunohistochemistry (IHC) for CLDN18 was performed on 29 cases included in the spatial transcriptomics cohort (4 nonmucinous adenocarcinomas and 5 IMAs) as well as on additional lung adenocarcinoma cases comprising 107 nonmucinous adenocarcinomas and 20 IMAs. CLDN18 and CLDN18.2 staining was performed using the VENTANA CLDN18 (43–14A) RxDx Assay (09620656001, Roche, Basel, Switzerland) and an anti‐CLDN18.2 antibody (EPR19202, 1:200 dilution; Abcam, Cambridge, UK), respectively. In a case showing high *HER2* mRNA expression on spatial transcriptomic analysis, HER2 protein expression was assessed by IHC using the PATHWAY anti‐HER‐2/neu (4B5) rabbit monoclonal primary antibody (05999570001, Roche). *HER2* gene status was further evaluated by silver *in situ* hybridization (SISH) using the VENTANA HER2 Dual ISH DNA Probe Cocktail (08314373001, Roche), following the manufacturer's protocol.

## Results

### Patient characteristics

Of the 26 patients, 15 were male and 11 were female. The mean age of the patients was 67 years (range: 40–86 years). Pathological diagnoses included four cases of nonmucinous TRU‐type adenocarcinoma, 10 cases of SCC, and 12 cases of IMA. Tumor staging was as follows: stage I in 16 patients, stage II in eight patients, and stage III and IV in one patient each. Lymph node metastasis was identified in two patients, both classified as N1. A smoking history was documented in 13 of 26 patients, including one of four patients with nonmucinous TRU‐type adenocarcinoma (25.0%), eight of 10 with SCC (80.0%), and four of 12 with IMA (33.3%). Genetic mutations were identified in a total of eight cases. In nonmucinous TRU‐type adenocarcinoma, one case harbored an *EGFR* exon 19 deletion and one case exhibited a *KRAS* (G12V) mutation. In SCC, a *KRAS* (G12V) mutation was detected in one case. Among the IMA cases, *KRAS* (G12V and G12D) mutations were detected in five out of 12 cases. A summary of these findings is provided in Table 1.

**Table 1:** Patients' clinicopathological characteristics. Values are presented as mean (range) or number (%).

Parameter	No. (%) or value
Age (yrs)	67 (40–86)
Sex	
Male	15 (57.7)
Female	11 (42.3)
Pathologic diagnosis	
Adenocarcinoma	4
Never smoker	3 (75.0)
Smoker	1 (25.0)
Squamous cell carcinoma	10
Never smoker	2 (20.0)
Smoker	8 (80.0)
Invasive mucinous adenocarcinoma	12
Never smoker	8 (66.7)
Smoker	4 (33.3)
Pathologic stage	
Nonmucinous adenocarcinoma (*n =* 4)	
I	4 (100.0)
Squamous cell carcinoma (*n =* 10)	
I, II	9 (90.0)
III, IV	1 (10.0)
Invasive mucinous adenocarcinoma (*n =* 12)	
I, II	11 (91.6)
III, IV	1 (8.3)
Genetic alterations	
Nonmucinous adenocarcinoma	2 (33.3)
*EGFR* (Exon 19 del)	1
*KRAS* (G12V)	1
Squamous cell carcinoma	1 (16.7)
*KRAS* (G12V)	1
Invasive mucinous adenocarcinoma	5 (41.7)
*KRAS* (G12V, G12D)	5

### Morphologic evaluation of the selected regions

Histological evaluation revealed a spectrum of morphological changes in the selected lesions (Figure 1; supplementary material, Figure S2). NB exhibited a pseudostratified ciliated epithelium with scattered club cells, maintaining uniform epithelial thickness and an intact basement membrane. The BM showed replacement of the normal alveolar lining by bronchiolar‐type cuboidal or columnar cells with preserved polarity, mild nuclear enlargement, and occasional mucinous cytoplasm, consistent with bronchiolization of the alveolar spaces. MM demonstrated a cuboidal or columnar epithelium with bluish mucins in the cytoplasm without cilia. The nuclei were located basally without atypia. MD is a hypothetical intermediate lesion, defined in this study as a mucinous epithelial proliferative lesion with nuclear atypia and architectural complexity, proposed to represent a morphologic continuum between metaplasia and carcinoma *in situ*. IMA exhibited confluent growth of tall columnar mucinous cells with basal nuclei and abundant intracytoplasmic and luminal mucins, forming lepidic and acinar patterns with variable stromal invasion. Nonmucinous TRU‐type adenocarcinomas consist of cuboidal cells that form acinar or papillary structures with eosinophilic cytoplasm and distinct nucleoli, recapitulating AT2 differentiation. SqM/D showed the transformation of the normal columnar or cuboidal epithelium into stratified squamous epithelium with distinct intercellular bridges and occasional keratinization. Nuclear atypia and increased mitotic activity were observed in some foci, indicating early dysplastic changes. SCC displays invasive nests or sheets of polygonal tumor cells with eosinophilic cytoplasm, prominent intercellular bridges, and keratin pearl formation. Depending on the tumor site, they were classified as pSCC that arose in the parenchyma and cSCC that arose in the bronchi.

**Figure 1:**
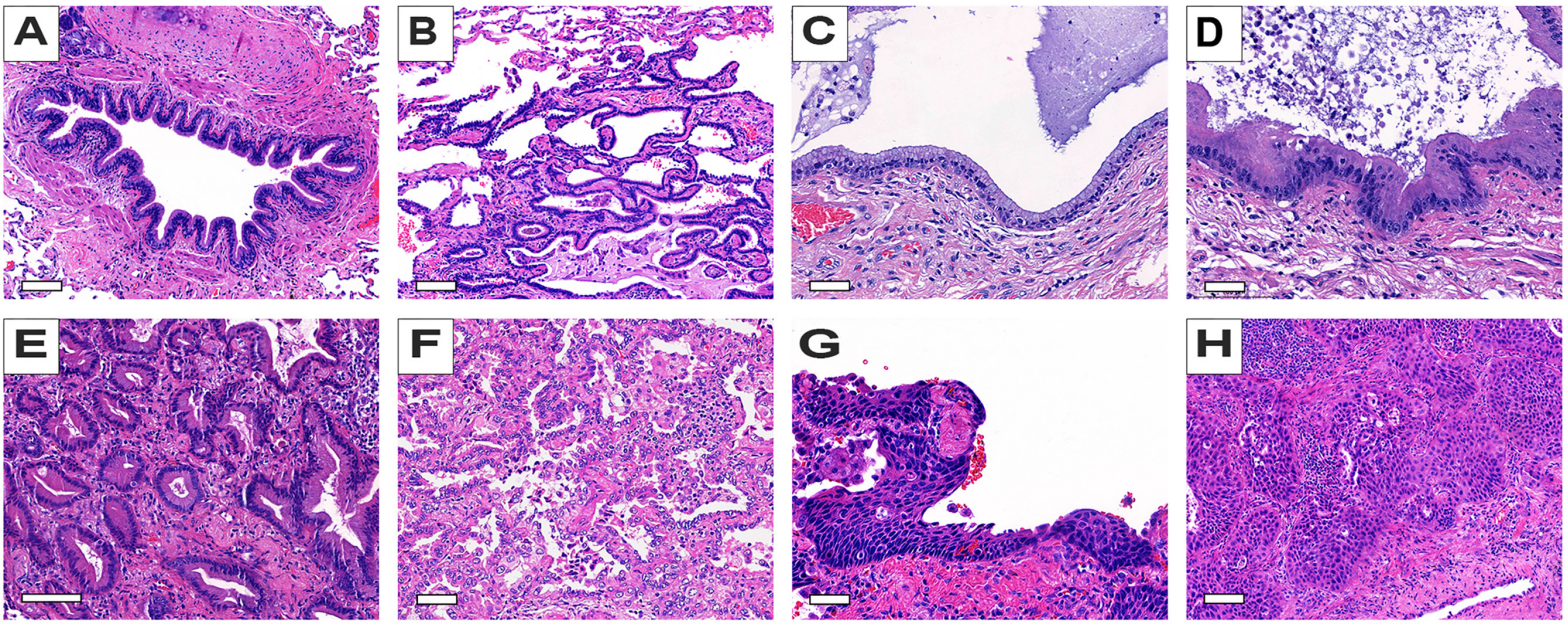
Histologic features of normal bronchiole (A), peribronchiolar metaplasia (B), mucinous metaplasia (C), mucinous dysplasia (D), IMA (E), nonmucinous terminal respiratory unit‐type adenocarcinoma (F), squamous metaplasia (G), and squamous cell carcinoma (H). Scale bars, panels A, B, E, F, 100 μm; panels C, D, G, H, 50 μm.

### Spatial transcriptomic profiling and integration

First, we applied principal component analysis, followed by UMAP analysis. The UMAP showed mixed spots on the slides, revealing that the batch effect was removed (Figure 2A). When plotted by group, MD and IMA were placed in a distinct cluster (Figure 2B). The MM and MD samples were mostly located in and around the IMA clusters. BM and NB samples were located between the two AdC clusters. Two public datasets (GSE200563 and GSE265899) were used for the expansion and validation. GSE265899 consists entirely of nonmucinous adenocarcinoma cases (confirmed through personal communication with the author). UMAP showed that samples from both datasets were mixed with nonmucinous adenocarcinomas, indicating that the integration worked well. Nonmucinous adenocarcinomas from this and two other studies were embedded in two distinct clusters (Figure 2B). InferCNV analysis demonstrated that mucinous lesions were distinct from other types of lesions and exhibited relatively stable CNV profiles. Progressive 17p loss was observed from the mucinous metaplasia to the IMA (Figure 2C).

**Figure 2:**
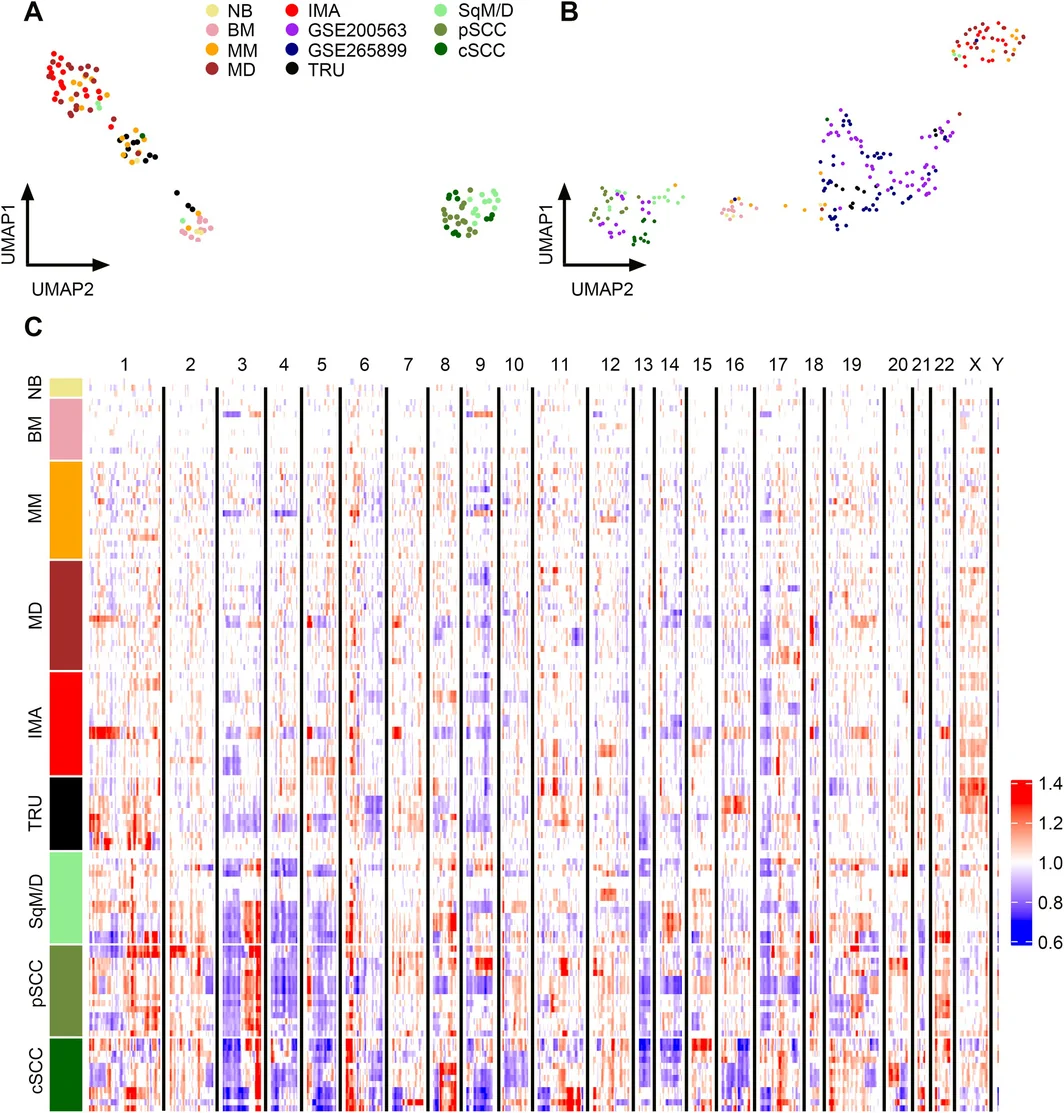
UMAP embedding and inferCNV analysis of spatial transcriptomic lesions. (A) UMAP projection of lesions profiled in this study. (B) UMAP generated from integrated dataset combining this study with two external cohorts (GSE200563 and GSE265899). (C) InferCNV heatmap derived from integrated data, illustrating copy‐number alterations in epithelial lesions relative to nonneoplastic references. InferCNV values represent modified expression normalized to reference ROIs, with a baseline centered at 1. UMAP, Uniform Manifold Approximation and Projection. NB, normal bronchiole; BM, bronchiolar metaplasia; MM, mucinous metaplasia; IMA, invasive mucinous adenocarcinoma; TRU AdC, terminal respiratory type adenocarcinoma; SqM/D, squamous metaplasia and dysplasia; pSCC, peripheral squamous cell carcinoma; cSCC, central squamous cell carcinoma.

### Unsupervised clustering and marker‐based annotation

Unsupervised clustering revealed six transcriptionally distinct clusters (0–5) representing the major epithelial and stromal lineages (Figure 3). Cluster‐defining marker genes are listed in supplementary material, Table S1, and the distribution of sample types across clusters is summarized in Table 2. Cluster 0 contained a mixture of GSE200563 and GSE265899 and was enriched for AT2‐associated markers, including *NAPSA*, *SFTA2*, and *NKX2‐1*. This cluster reflected an alveolar‐type adenocarcinoma program characterized by preserved surfactant‐related transcriptional activity. Cluster 1 was composed predominantly of GSE200563, with additional cSCC cases, and expressed basal/squamoid markers, such as *KRT5*, *TP63*, and *NTRK2*. This profile supported a basal/squamoid epithelial identity, consistent with transitional states toward squamous differentiation. Cluster 2 was highly specific for IMA and demonstrated strong expression of mucinous/gastric lineage genes, including *MUC5AC*, *MUC6*, *TFF1*, *TFF2*, *CLDN18*, *HNF4A*, and *ANXA10*. This cluster corresponds to an IMA and recapitulates the previously described gastric‐type differentiation signature [5]. The expression of representative cluster‐defining genes across lesion types is further illustrated by violin plots (Figure 4). Notably, *CLDN18* mRNA expression was markedly increased in IMAs, prompting immunohistochemical validation of CLDN18 protein expression. In total, 136 lung adenocarcinomas were examined, including 111 nonmucinous adenocarcinomas and 25 IMAs. Strong, diffuse membranous CLDN18 expression in nearly all tumor cells was observed in 23 of 25 IMAs, whereas nonmucinous adenocarcinomas showed either complete absence of staining or only focal, weak membranous expression in a small subset of tumor cells (Figure 3; supplementary material, Figure S3). Using a CLDN18.2‐specific antibody, CLDN18.2 expression was confirmed in IMAs but was absent in nonmucinous adenocarcinomas (supplementary material, Figure S3). In one patient, markedly increased *ERBB2* transcript levels were associated with strong HER2 protein expression, and *ERBB2* gene amplification was confirmed by silver *in situ* hybridization (Figure 5). Cluster 3 was largely composed of GSE200563 cases and showed enrichment of *CALCA*, *PCSK1/2*, and *CALB1*, representing a neuroendocrine‐associated transcriptional program within a subset of adenocarcinoma cells. Cluster 4 was enriched for BM also including four MMs and showed high expression of club and ciliated cell markers, including *C20orf85 (CIMP1)*, *SCGB1A1*, *FOXJ1*, and *TPPP3*, which is consistent with the bronchiolar lineage program. Cluster 5 showed a heterogeneous gene expression profile without a clear lineage‐specific signature, including diverse genes related to multiple biological processes.

**Figure 3:**
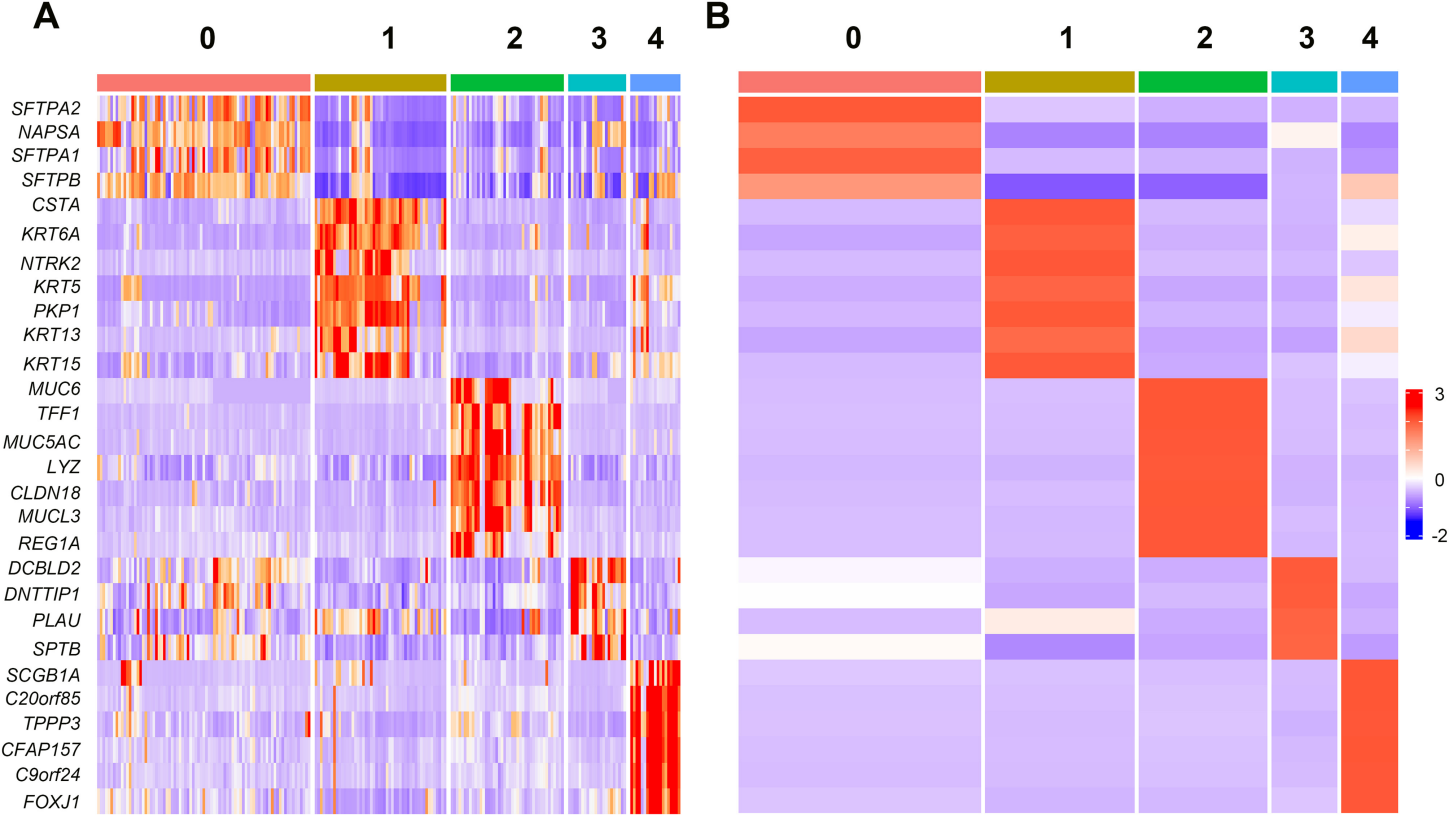
Results of clustering analysis. (A) Heatmap showing expression of top cluster‐defining genes across individual ROIs, arranged by cluster. (B) Heatmap summarizing mean expression of these genes within each cluster, highlighting cluster‐specific expression patterns. Cluster 5 is omitted, as it does not exhibit a lineage‐specific gene expression pattern.

**Table 2:** Distribution of specimen types across transcriptional clusters.

Specimen type	Cluster					
	0	1	2	3	4	5
GSE200563	27	14	0	14	0	9
GSE265899	37	0	1	7	1	2
BM	1	0	0	0	9	0
cSCC	0	11	0	1	0	0
IMA	0	0	17	0	0	0
MD	1	0	16	0	0	1
MM	5	0	7	0	4	0
NB	1	0	0	0	0	2
pSCC	0	15	0	0	0	0
SqM/D	0	10	2	0	3	0
TRU AdC	9	0	0	0	0	3

BM, bronchiolar metaplasia; cSCC, central squamous cell carcinoma, IMA, invasive mucinous adenocarcinoma; MD, mucinous dysplasia; MM, mucinous metaplasia; NB, normal bronchiole; pSCC, peripheral squamous cell carcinoma; SqM/D, squamous metaplasia and dysplasia; TRU AdC, terminal respiratory type adenocarcinoma.

**Figure 4:**
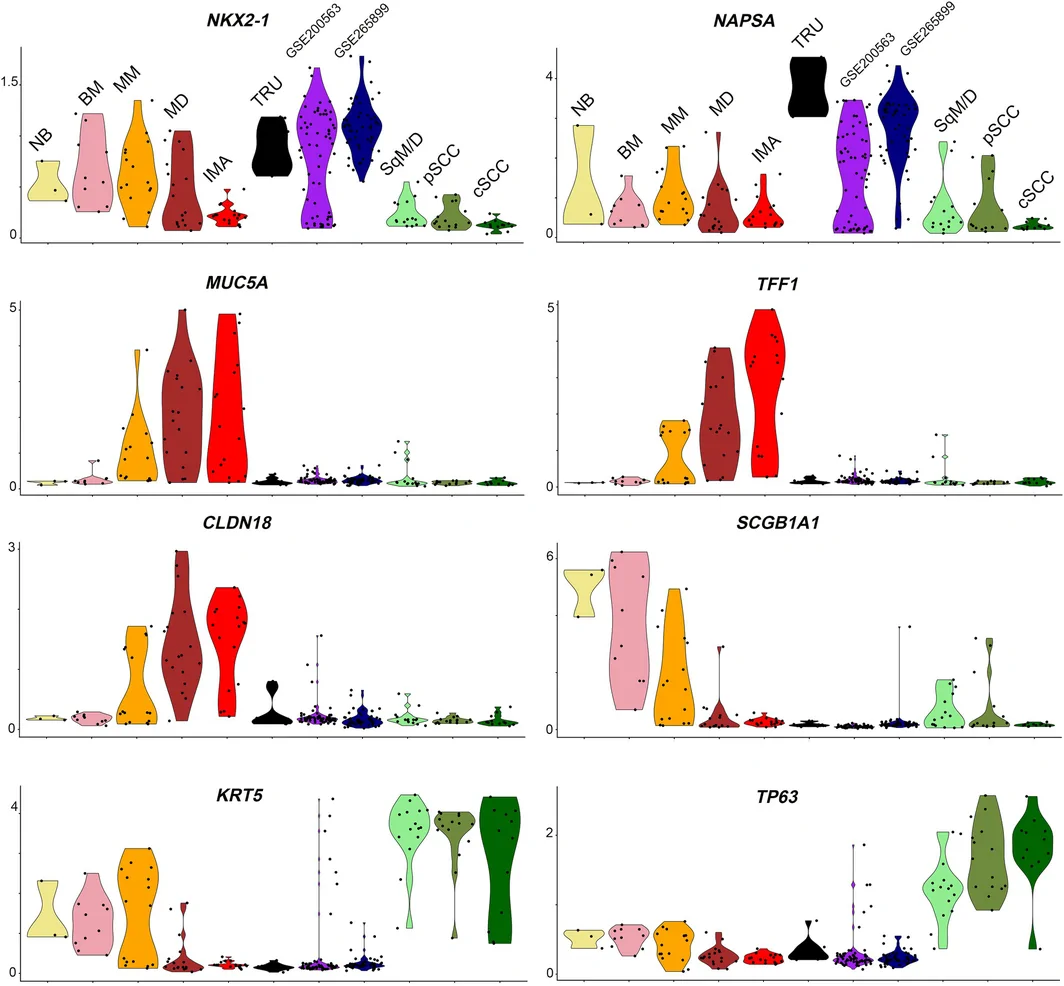
Violin plots demonstrating the spatial distribution and expression levels of representative cluster‐defining genes across lesion types. NB, normal bronchiole; BM, bronchiolar metaplasia; MD, mucinous dysplasia; IMA, invasive mucinous adenocarcinoma; TRU AdC, terminal respiratory‐type adenocarcinoma; SqM/D, squamous metaplasia and dysplasia; pSCC, peripheral squamous cell carcinoma; cSCC, central squamous cell carcinoma.

**Figure 5:**
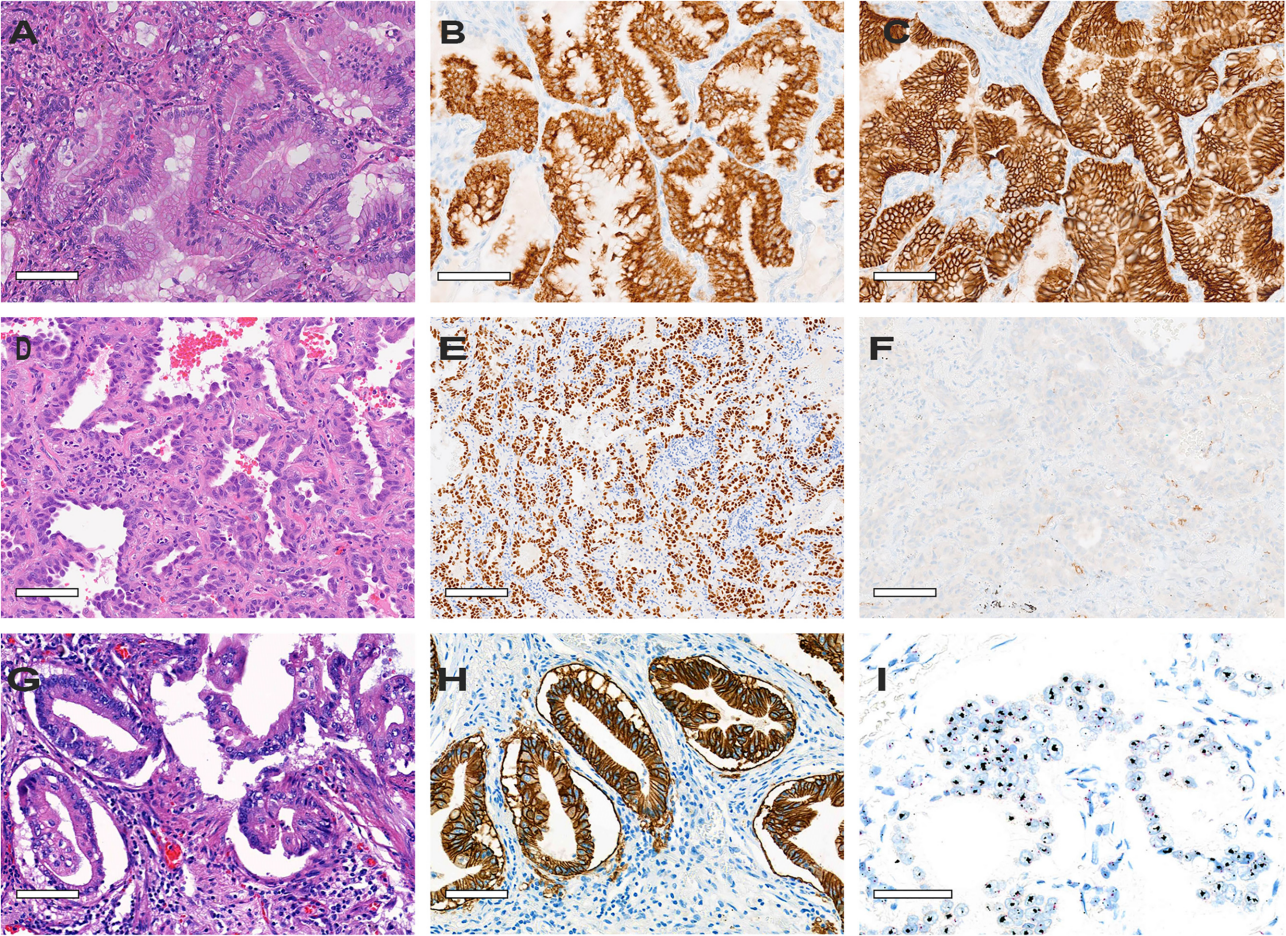
Claudin18 and HER2 expression. (A) IMA shows characteristic mucinous morphology. (B and C) The tumor cells demonstrate uniformly strong expression of MUC5AC (B) and CLDN18 (C). (D) In contrast, nonmucinous terminal respiratory unit‐type adenocarcinomas exhibit typical acinar architecture. (E) Tumor cells retain diffuse nuclear TTF‐1 expression. (F) They are negative for CLDN18. (G–I) One IMA expressed HER2 (H), and the gene amplification is demonstrated using silver *in situ* hybridization (I). Scale bars, panels A, B, C, D, F, G, H, 100 μm; panel E, 200 μm; panel I, 50 μm.

### Club cells as a probable origin of the mucinous cluster

Integration of our dataset with two external cohorts and projection onto the HLCA via Seurat label transfer identified lineage associations across bronchiolar and tumor evolution (Table 3). BMs and NBs were predominantly mapped to multi‐ciliated cells, indicating preservation of normal epithelial cells. MMs and MDs exhibited mixed contributions from club and goblet cells with minor multi‐ciliated and suprabasal inputs, whereas the IMA was enriched in club and goblet identities, consistent with its mucinous phenotype. Conventional adenocarcinomas (TRU, GSE200563, and GSE265899) were largely assigned to AT2 cells, supporting an alveolar lineage origin. GSE200563 showed additional hillock‐like and suprabasal contributions, while GSE265899 retained low‐level goblet and multi‐ciliated signals. pSCCs and cSCCs were predominantly mapped to the suprabasal and hillock‐like lineages, with pSCC and cSCC showing particularly high suprabasal proportions, reflecting progressive squamous differentiation.

**Table 3:** Predicted epithelial cell identities based on Human Lung Cell Atlas label transfer.

	AT0	AT2	Club	Goblet	Hillock‐like	Multiciliated	Suprabasal
NB	0	1	0	0	0	2	0
BM	0	0	0	0	0	10	0
MM	0	3	7	1	0	4	1
MD	0	1	11	6	0	0	0
IMA	0	1	9	6	0	0	1
TRU AdC	0	12	0	0	0	0	0
GSE200563	0	37	0	3	15	0	9
GSE265899	1	41	0	5	0	1	0
SqM/D	0	0	0	2	4	0	9
pSCC	0	0	0	0	3	0	12
cSCC	0	0	0	0	4	0	8

NB, normal bronchiole; BM, bronchiolar metaplasia; MM, mucinous metaplasia; IMA, invasive mucinous adenocarcinoma; TRU AdC, terminal respiratory type adenocarcinoma; SqM/D, squamous metaplasia and dysplasia; pSCC, peripheral squamous cell carcinoma; cSCC, central squamous cell carcinoma.

## Discussion

In this study, we measured mRNA expression across 118 carefully selected ROIs using the GeoMx spatial transcriptomics platform. Although the resolution was lower than that of scRNA‐seq, each ROI was exclusively composed of cytokeratin‐positive epithelial cells and represented a morphologically homogeneous population. This approach estimated the single‐cell‐like specificity and preserved the architectural context of the lesions.

The gene expression patterns observed across the mucinous lesions were highly informative. Club cell‐associated markers, such as *SCGB1A1* and *MUC5B*, were prominent in MM and diminished with progression to MD and IMA. Conversely, genes associated with gastric differentiation (*TFF1*, *TFF2*, *ANXA10*, *REG4*, *FER1L6*, and *MUC5AC*) showed a progressive increase, peaking in IMA. This reciprocal pattern strongly suggests that MM cells, initially retaining a club cell phenotype, gradually acquire goblet cell‐like features and ultimately assume a gastric‐type transcriptional program characteristic of IMA. Therefore, the inverse expression of *SCGB1A1* and *MUC5B* and genes associated with gastric differentiation may represent a molecular signature of this transition. Importantly, these observations are consistent with recent scRNA‐seq studies, demonstrating that goblet cells may emerge from club cells [11, 12]. Our label‐transfer analysis of HLCA further corroborates this trajectory. In the IMA, the predicted cell types were primarily club (nine ROIs) and goblet cells (six ROIs), with occasional AT2 and suprabasal identities (one ROI each). Among the 16 MM spots from the nine patients, the majority were predicted as club cells (seven ROIs), with additional annotations as multi‐ciliated (four ROIs), goblet (one ROI), AT2 (three ROIs), and suprabasal (one ROI) cells. This broader diversity likely reflects the presence of residual ciliated cells and type II pneumocytes that have not yet fully undergone a metaplastic transition. In contrast, 18 MD spots from eight patients were more uniform, with most classified as club (11 ROIs) or goblet (six ROIs) cells and only a single AT2 annotation. Taken together, these findings indicate that, while MM represents a transitional stage with heterogeneous cellular identities, MD and IMA are predominantly composed of cells whose transcriptional features most closely resemble those of club and goblet cells, supporting the proposed progression model.

In contrast, across 124 ROIs derived from three independent datasets (TRU, GSE200563, and GSE265899), 91 were predicted to be AT2 cells, with smaller subsets annotated as goblet (eight ROIs), multi‐ciliated (one ROI), hillock‐like (15 ROIs), and suprabasal (nine ROIs) cells. The strong predominance of AT2 annotations aligns with the established role of AT2 cells as the cells of origin of nonmucinous TRU‐type adenocarcinomas, supporting the robustness of the analysis. Consistent with this, AT2‐associated markers, such as *SFTPA* and *SFTPB*, were enriched, further underscoring their alveolar lineage. Notably, the presence of neuroendocrine‐related transcripts in the selected clusters indicated molecular heterogeneity, a feature frequently encountered by pathologists in clinical practice.

SCC typically arises in the bronchial mucosa through squamous metaplasia induced by extrinsic factors, such as smoking. SCC may also develop in the lung periphery, where bronchiolization precedes squamous metaplasia. We hypothesized that pSCCs share bronchiolization and cellular origins with IMA. Among the 42 squamous lesions analyzed, 40 ROIs were predicted to originate from suprabasal or hillock‐like cells, whereas two ROIs of squamous metaplasia were predicted to originate from goblet cells. We assumed that the squamous metaplasia was derived from columnar cells in the bronchiolized alveoli. However, once the morphology shifts to the squamous phenotype, the gene expression profile resembles that of squamous cells. Consequently, an early columnar cell gene signature was not detected in these lesions. Taken together, our label transfer results seem to be very reliable in that nonmucinous TRU‐type adenocarcinoma has been predicted to be AT2 cells, and squamous cell carcinoma has been predicted to be suprabasal or hillock cells.

To clarify the potential club cell origin of IMA, IHC was performed for club cell markers (SCGB1A1, SPDEF, NKX3‐1, MUC5AC, and MUC5B), which were identified by comparing club and other epithelial cells in the HLCA database. Although the gene expression of these markers was increased throughout MM, MD, and IMA, the protein was not detected, except for MUC5AC and MUC5B, expressed in MD and IMA. However, the discordance between RNA abundance and protein detection remains unresolved. Recent transcriptomic‐proteomic studies have shown that such discrepancies are common and may arise from multiple biological layers of regulation, including transcriptional bursting, variable mRNA stability, translational efficiency, and differences in protein half‐life [21, 22, 23]. Early mucinous transformation may amplify these effects and produce transcripts without an immediate or proportional protein output. Technical factors, such as epitope accessibility, fixation‐related antigen loss, and limited antibody sensitivity, may further obscure the true protein levels. Although the transcriptional profile supports a club‐like trajectory, confirmation of protein levels remains challenging. Future studies incorporating mass spectrometry‐based proteomics or high‐plex spatial proteogenomic platforms will be essential to validate these transcript‐level findings at the protein level and further clarify club cell involvement in IMA development.

Six distinct clusters were identified by cluster analysis. Notably, all 17 IMA ROIs and 16 of the 18 mucinous dysplasia ROIs were grouped into cluster 2. The top 10 upregulated genes in this cluster were *MUC6*, *TFF1*, *MUC5AC*, *TFF2*, *XCR1*, *LYZ*, *CLDN18*, *MUCL3*, *TSPAN7*, and *REG1A*. *CLDN18.1* is typically expressed in the lung, whereas *CLDN18.2* is gastric‐specific; therefore, the strong immunohistochemical expression of CLDN18 in IMA suggests that this signal is derived from *CLDN18.2*, as supported by our additional staining using a CLDN18.2‐specific antibody and by findings from a recently published study [24]. CLDN18 IHC was performed showing that CLDN18 was strongly expressed in IMAs but not in nonmucinous TRU‐type adenocarcinoma. Collectively, this gene signature indicates a program of gastric differentiation, highlighting the distinct molecular phenotypes of mucinous lesions. Importantly, CLDN18 expression identified in IMA may have potential therapeutic implications. Recent development of CLDN18.2‐targeted therapies in other malignancies raises the possibility that a subset of IMA patients could benefit from similar approaches [24, 25, 26].

InferCNV analysis revealed that MM, MD, and IMA displayed relatively stable CNV profiles compared to that of other lesions. These three mucinous entities exhibited substantially similar CNV patterns, underscoring their close biological relationship. Importantly, 13q deletion, a recurrent alteration in NSCLC [27, 28], was absent in mucinous lesions, whereas progressive 17p loss, including the *TP53* locus, was observed from the MM to the IMA. Despite the small sample size, these findings provide novel insights into the pathogenesis of IMA and merit further validation.

To our knowledge, this is the first study to apply spatial transcriptomics to IMAs. By integrating GeoMx spatial transcriptomic data with two external cohorts and HLCA, we revealed a continuum of transcriptional alterations, suggesting a shift from club‐cell‐associated programs toward goblet‐ and gastric‐type signatures that ultimately define IMA. The nonmucinous TRU‐type adenocarcinomas from our dataset and the two external datasets consistently clustered together on UMAP and were predicted to originate from AT2 cells, whereas IMAs demonstrated strong CLDN18 expression with IHC, which was consistent with their RNA‐level upregulation. These convergent findings support the robustness and reliability of the analytical approach. The long‐standing assumption that nonmucinous TRU‐type adenocarcinomas arise from either AT2 or club cells has been widely studied but remains inadequately supported by direct evidence. Our results indicate that the prevailing model warrants re‐evaluation. This study had several limitations. First, the spatial resolution of spatial transcriptomics is lower than that of scRNA‐seq, which limits the ability to perform robust trajectory analysis. Second, protein‐level validation of club‐cell‐associated transcripts was not achievable with the antibodies tested. Future work incorporating proteomics and scRNA‐seq will be crucial to determine whether club cells truly undergo metaplastic transitions toward the mucinous lineage and contribute to the development of IMA.

## Author contributions statement

DHS designed this study and analyzed the results. JYP and DYK performed experiments and wrote the manuscript. HRP secured the funding for the study. JHL and HJL critically reviewed and edited the manuscript. All authors have read and approved the final paper.

## Supporting information


**Figure S1.** Representative ROIs selected for GeoMx digital spatial profiling
**Figure S2**. Histological spectrum of mucinous lesions
**Figure S3**. Claudin 18 expression

**Table S1.** Cluster‐defining marker genes identified by unsupervised clustering

## Data Availability

Raw and processed data are deposited at GEO under accession GSE317545. Public datasets used include GSE200563 (https://www.ncbi.nlm.nih.gov/geo/query/acc.cgi?acc=GSE200563) and GSE265899 (https://www.ncbi.nlm.nih.gov/geo/query/acc.cgi?acc=GSE265899).
