# Supplementary material for: Spatial transcriptomics reveals club cell features and strong CLDN18 expression along the mucinous pathway to invasive mucinous adenocarcinoma
Source: J Pathol. 2026 Sep 8;270(4):497–506. doi: 10.1002/path.70120 (PMC13728818; doi:10.1002/path.70120)
Supplement: Supplementary file 1 [file PATH-270-497-s002.docx]

**Spatial transcriptomics reveals club cell features and strong CLDN18 expression along the mucinous pathway to invasive mucinous adenocarcinoma**

JY Park *et al J Pathol.* <https://doi.org/10.1002/path.70120>

**Supplementary Figures S1–S3
Supplementary Table S1** (provided as a separate Excel sheet)

**Figure S1.** **Representative ROIs selected for GeoMx digital spatial profiling.** To define ROIs, two consecutive sections were prepared from each tissue block. ROIs were initially selected on H&E-stained sections (A and C) by two pathologists through consensus review, and the corresponding regions were subsequently identified on the immunofluorescence slides (B and D) for GeoMx DSP analysis. Scale bars, 2 and 5 mm.

**Figure S2. Histological spectrum of mucinous lesions.** (A–C) Mucinous metaplasia, composed of mucin-producing columnar cells with interspersed ciliated cells and an intact basal cell layer, typically involving bronchioles or areas of bronchiolar metaplasia. (D–F) Mucinous dysplasia showing a more extensive proliferation of mucinous cells with enlarged, rounded nuclei, increased nuclear overlap, irregular nuclear positioning, and partial loss of polarity. (G–H) Invasive mucinous adenocarcinoma demonstrating gland-forming mucinous epithelium lacking both ciliated and basal cells, consistent with loss of normal airway differentiation. In some areas, intracellular mucin is reduced, resulting in eosinophilic cytoplasm. Scale bars, 50 µm.

**Figure S3.** **Claudin 18 expression.** Invasive mucinous adenocarcinoma (A and D) shows strong and diffuse circumferential membranous expression of CLDN18 using the CLDN18 (43-14A) antibody (B and E). Staining with a CLDN18.2-specific antibody (EPR19202) confirmed that the expression was attributable to CLDN18.2 (C and F). In contrast, nonmucinous adenocarcinoma (G) showed only focal and partial membranous staining in a small subset of tumor cells with the CLDN18 (43-14A) antibody (H) and was completely negative for CLDN18.2 (I). Scale bars, panels (A), (B), (E), 200 $\mu m$; panels (C), (F), (H), (I), 50 µm; panels (D) and (G), 100 µm.
